# Clinical guidelines for managing menopausal symptoms in women with (a history of) breast cancer

**DOI:** 10.52054/FVVO.15.4.102

**Published:** 2023-12-13

**Authors:** J Servayge, A.C. Verduyn, A Page, L Lagaert, W.A.A. Tjalma

**Affiliations:** Department of Gynaecology, University Hospital Ghent, 9000 Ghent, Belgium; Department of Gynaecology, CHU Saint Pierre, 1000 Brussels, Belgium; Department of Gynaecology, AZ Sint-Elisabeth Zottegem, 9620 Zottegem, Belgium; Multidisciplinary Breast Clinic, Gynecological Oncology Unit, Department of Obstetrics and Gynecology, Antwerp University Hospital, University of Antwerp, Drie Eikenstraat 655, 2650 Edegem, Belgium

**Keywords:** Menopause, symptoms, breast cancer, menopausal symptoms, hormone replacement therapy, tamoxifen, aromatase inhibitors

## Abstract

**Background:**

One in eight women will be diagnosed with breast cancer. At the time of diagnosis, 75% of patients are postmenopausal. Many will receive anti-hormone therapy, which often induces menopausal symptoms. Premenopausal breast cancer patients frequently become postmenopausal as a result of the treatment and often experience menopausal symptoms. The increased incidence of breast cancer, combined with longer survival, has led to an increase in the number of women experiencing menopausal symptoms. Therefore, the management of menopausal symptoms in women with a history or current breast cancer is a relevant and common clinical problem.

**Objectives:**

To provide a clinically useful overview of the steps in the management of menopausal symptoms in women with (a history of) breast cancer.

**Materials and Methods:**

A comprehensive literature review was conducted by authors JS and WT using the PubMed and Medline databases. Abstracts were critically appraised and, where appropriate, the full text was analysed.

**Main outcome measures:**

Not applicable.

**Results:**

Depending on the condition, either meta-analyses, randomised controlled trials or retrospective cohorts were identified. No evidence was found for some proposed treatments.

**Conclusions:**

Menopausal symptoms in women with (a history of) breast cancer require a patient-tailored approach. Shared decision making is paramount and adequate up-to-date knowledge can help the breast cancer specialist to advise and guide patients accordingly.

**What is new?:**

A comprehensive, clinically-based overview of evidence-based treatment options for menopausal symptoms in women with (a history of) breast cancer.

## Introduction

In Belgium, 11,000 women are diagnosed with breast cancer every year, according to the Belgian Cancer Registry. One in eight women will develop breast cancer during her lifetime and almost 75% of patients are postmenopausal at the time of diagnosis. The increase in incidence and survival due to early detection and improved treatment protocols is prolonging the survival of premenopausal women and consequently increasing the number of women experiencing climacteric symptoms. Therefore, the treatment and management of menopausal symptoms in women with a history or current breast cancer is a relevant and common clinical problem (Franzoi et al., 2021). Table I lists menopausal symptoms, treatment options, grade of recommendation and level of evidence.

**Table I:** Summary of recommendations.

Recommendation		Grade of recommendation	Level of Evidence
Vasomotor symptoms			
Lifestyle			
Smoking cessation		Strongly recommended	++++ (1)
Restrict alcohol intake		Strongly recommended	++++ (1)
Body weight BMI 20-25		Strongly recommended	++++ (1)
Supplementation		Strongly recommended	++++ (1)
Physical exercise		Strongly recommended	++++ (1)
Alternative treatment			
	Education, counselling, yoga, mindfulness, CBT	Strongly recommended	++++ (1)
	Acupuncture	Recommended	++++ (1)
	Qi Gong or Tai Chi	Optional	++++ (1)
Intermittent cessation of endocrine therapy		Optional	+++ (2)
Systemic non-hormonal treatment			
Clonidine		Recommended	+++ (2)
SSRI-SNRI			
	Paroxetine	Strongly recommended	++++ (1)
	Citalopram	Recommended	++++ (1)
	Escitalopram	Recommended	++++ (1)
	Venlafaxine	Recommended	++++ (1)
	Fluoxetine	Discouraged	++++ (1)
	Sertraline	Discouraged	++++ (1)
Gabapentin		Recommended	++++ (1)
Oxybutynin		Recommended	++++ (1)
Systemic hormonal replacement therapy		Strongly discouraged	++++ (1)
Tibolone		Strongly discouraged	++++ (1)
Arthralgie			
Supplementation		Discouraged	++++ (1)
Body weight and physical exercise		Strongly recommended	++++ (1)
Acupuncture		Recommended	++++ (1)
Duloxetine		Recommended	++++ (1)
Sleeping disorders			
Sleep hygiene		Strongly recommended	+++ (2)
Melatonin		Strongly recommended	++++ (1)
CBT		Strongly recommended	++++ (1)
Tai Chi and acupuncture		Recommended	+++ (2)
Vaginal dryness and dyspareunia			
Sexual counselling, psychosexual education, couples therapy		Strongly recommended	++ (3)
Topic vaginal non-hormonal treatment		Strongly recommended	++++ (1)
Topic vaginal hormonal treatment			
	Estriol (E3)	Recommended	++++ (1)
	DHEA	Optional	+++ (2)
	Ospemifene	Strongly discouraged	++ (3)
Osteopenia/ osteoporosis			
Vitamine D and Calcium		Strongly recommended	++++ (1)
Bisphosphates		Strongly recommended	++++ (1)

## Search strategy

A PubMed search was conducted from 01/01/2000 to 01/10/2023 with the following keywords: “menopause”, “symptoms”, “breast cancer”, “management”, “endocrine therapy”, “aromatase inhibitors”, “tamoxifen”, “vasomotor symptoms”, “arthralgia”, “sleeping disorders”, ”genitourinary symptoms”, “osteopenia”, “osteoporosis”, “hormone replacement therapy”, “non-hormonal treatments”, “SSRI”, “SNRI”, “clonidine”, “gabapentin” and “vaginal oestrogen. Abstracts were critically appraised and, where appropriate, the full text was analysed.

To select participating authors, members of the Special Interest Group Senology of the Flemish Society of Obstetrics and Gynecology were contacted during a quarterly meeting and by an e-mail. Doctors expressing their interest were selected after e-mail contact with W.A.A. Tjalma. J. Servayge was selected first author because of his major contribution to this article. Authors A-C Verduyn, A.L. Page and L. Lagaert provided insight and added some paragraphs on “vaginal dryness and dyspareunia” and “systemic non-hormonal treatment of vasomotor symptoms”. The content of the final manuscript was presented in PowerPoint-format at the quarterly meeting of the Flemish Society of Senology. Society members provided input during the presentation, and this was also added in the text.

## Evidence

### Vasomotor symptoms

Vasomotor symptoms (VMS) occur in 65-85% of breast cancer patients (Tao et al., 2017). They are described as a temporary sensation of warmth over the skin, profuse sweating, flushing of the chest and face, often accompanied by sweating, dizziness, nausea, chills, fatigue and sleep disturbance. Symptoms can last for years and have a significant impact on social functioning, work, enjoyment of life and overall quality of life.

In women without (history of) breast cancer who experience menopausal symptoms, hormone replacement therapy (HRT) has been shown to have a positive effect on quality of life (Franzoi et al., 2021). Non-pharmacological treatments such as lifestyle adjustments, SSRIs, or oxybutynin are less effective (Tao et al., 2017; Tran et al., 2021; Duncan et al., 2017). Two large randomised controlled trials show that systemic HRT is contraindicated in women with breast cancer (or a history of breast cancer) (Holmberg et al., 2004; Fahlen et al., 2013). A suitable alternative for the treatment of menopausal symptoms in this growing group of patients is therefore needed.

#### Lifestyle

##### Smoking cessation

Several cross-sectional studies indicate a significantly increase in frequency and severity of vasomotor symptoms in smokers (Greendale and Gold, 2005). A longitudinal analysis showed that women who had quit smoking for more than 5 years had significantly fewer and milder vasomotor symptoms (Smith et al., 2015).

##### Alcohol intake

The relationship between alcohol consumption and the risk of vasomotor symptoms is controversial. Observational studies show mixed results and suggest that 1 unit of alcohol per day does not influence the occurrence of vasomotor symptoms (Avis et al., 2018; Kwon et al., 2022; Greendale and Gold, 2005). There are no data on the effect of alcohol cessation on vasomotor symptoms in women with (a history of) breast cancer.

##### Body weight and physical activity

A randomised controlled trial by Huang et al. showed a significant improvement in hot flush frequency in women with a BMI between 25 and 50 (mean BMI 37) following an intensive weight loss programme (Huang et al., 2010). Independent of weight loss, exercise did not have a direct, significant beneficial effect on vasomotor symptoms. However, exercise has been shown to have a positive effect on general health (Huang et al., 2010; Boraz et al., 2001). There are no data on the effect of a BMI < 20 on the frequency and severity of hot flushes.

##### Supplementation

Many over-the-counter preparations are available with plant-based constituents that have weak oestrogenic activity. Supplementation with antioxidants, wild yam, black cohosh, phytoestrogens, soya sprouts (soya isoflavones), lignans (from linseed) and red clover has not been shown to have an effect on vasomotor symptoms in women with (a history of) breast cancer (Greenlee et al., 2009; Pruthi et al., 2012). This is in contrast to randomised trials where isoflavones seem to have a beneficial effect on vasomotor symptoms in women without (history of) breast cancer (Barnard et al., 2023; Chen and Chen, 2021). Long-term treatment with phytoestrogens has shown a statistically significant increase in the incidence of endometrial hyperplasia (Lethaby et al., 2013).

The efficacy of vitamin E has been investigated and compared with gabapentin in breast cancer survivors, but vitamin E produced only a marginal, statistically insignificant improvement in vasomotor symptoms (Biglia et al., 2009).

None of these proposed preparations are an acceptable alternative for the treatment of climacteric symptoms in women with (a history of) breast cancer (Kim et al., 2015).

Sérélys® is a non-hormonal dietary supplement consisting of, among other things, purified cytoplasmic extracts of pollen. There is currently insufficient data on the effect of Sérélys ® on hot flushes. Currently Sérélys cannot be recommended based on limited evidence (Seeger et al., 2018). A randomised controlled trial by Munstedt et al concluded that honey and bee pollen alleviated vasomotor symptoms. However, an increase in serum oestradiol levels was observed (Munstedt et al., 2015).

##### Alternative treatment

###### Educational, counselling, yoga, mindfulness, and cognitive-behavioural therapy

Randomized studies in women with (a history of) breast cancer have already demonstrated the beneficial effect of a yoga program (Carson et al., 2009), cognitive-behavioural therapy alone, and in combination with exercise (Duijts et al., 2012; Tran et al., 2021). There are mixed results regarding the effects of stress management, mindfulness, relaxation, and breathing techniques (Hartman et al., 2018; Lahart et al., 2018; Goldstein et al., 2017; Haller et al., 2017; Koch et al., 2017; Mann et al., 2012; Stefanopoulou and Grunfeld, 2017; van Driel et al., 2019).

###### Acupuncture

A systematic review by Pan et al. (2018) suggests that acupuncture may be an appropriate alternative treatment for hormone therapy-related side effects in breast cancer patients (Befus et al., 2018). However, they emphasize the lack of large-sample, multicentre, prospective randomized trials. A Cochrane review on the effects of acupuncture in alleviating vasomotor symptoms found no significant difference between acupuncture and sham acupuncture (Dodin et al., 2013; Chien et al., 2017; Chiu et al., 2016). However, there are 4 trials that showed a significant improvement in reducing the intensity and frequency of vasomotor symptoms with acupuncture compared to no treatment.

###### Qi Gong or Tai Chi

A meta-analysis by Luo et al., involving 15 articles and a total of 885 breast cancer patients,concludes that Tai Chi Chuan is effective in improving shoulder function, muscle strength, anxiety, and overall quality of life (Luo et al., 2020). This is supported by a meta-analysis by Li et al. (2023), although they add that the studies have low methodological quality and there is a need for larger randomized studies with long-term follow-up.

The effect of Qi Gong is less well studied, but there are randomised trials with small study groups that conclude that Qi Gong is effective in improving cancer-related fatigue. The added value and effects of Tai Chi/Qi Gong have been the subject of several randomised trials, such as the MATCH trial (Carlson et al., 2017). The effect of Tai Chi Chuan/Qi Gong on vasomotor symptoms has not been studied.

##### Systemic non-hormonal treatment

###### Clonidine

Clonidine has central α-adrenergic activity and may be used in the treatment of hypertension. Clonidine reduces vasomotor symptoms. Side effects may include drowsiness and dry mouth. Start with 0.05-0.075 mg twice daily. Evaluate after 4 weeks and discontinue if there is no improvement or if there are side effects. If treatment is stopped, it should be tapered gradually to avoid a rebound increase in blood pressure (Drewe et al., 2015; Boekhout et al., 2011; Friedman et al., 2011).

###### SSRI/SNRI

Selective Serotonin Reuptake Inhibitors (SSRIs) are a class of antidepressants but can be used for the treatment of hot flashes (Shams et al., 2014). They have also been shown to be safe in women with (a history of) breast cancer (Chubak et al., 2016). Paroxetine is the only non- hormonal therapy approved specifically for hot flashes in the United States (Simon et al., 2013). It inhibits the CYP2D6 enzyme, which also metabolizes tamoxifen into its active metabolite. Theoretically, this leads to a decrease in the active metabolite of tamoxifen. Simultaneous use of paroxetine and tamoxifen should, therefore, be avoided. However, this is contradicted by Haque et al. They looked at the recurrence of breast cancer in a retrospective cohort of 8099 women who used paroxetine and tamoxifen concurrently. No difference was found compared to the group that used tamoxifen alone (Haque et al., 2016). Venlafaxine and (es)citalopram have virtually no inhibitory effect on CYP2D6 and can be used. Citalopram and escitalopram alleviate hot flashes to a similar extent as paroxetine (Barton et al., 2010; Kalay et al., 2007). Fluoxetine and sertraline are less effective (Loprinzi et al., 2009).

Venlafaxine is a SNRI (Serotonin and Norepinephrine Reuptake Inhibitor) and inhibits the reuptake of both serotonin and norepinephrine.

At low doses (<150 mg/day) it acts like an SSRI. A randomised study by Bordeleau et al. (n=66) shows that venlafaxine (dose week 1: 37.5 mg once daily, dose weeks 2-4: 75 mg once daily) significantly reduces vasomotor symptoms (Bordeleau et al., 2010; Ramaswami et al., 2015; Boekhout et al., 2011). A study by Loprinzi et al. shows a 60% reduction in vasomotor symptoms after 4 weeks of treatment with venlafaxine 75 mg once daily (Loprinzi et al., 2000). The most common side effects of venlafaxine (SNRI) are dry mouth, nausea, decreased appetite, decreased libido, and constipation.

###### Gabapentin

Gabapentin was developed for the treatment of epileptic seizures but is also used for neuropathic pain and off-label for chronic low back pain or radicular pain. It may also be used to relieve hot flushes. It can be taken as a single dose at bedtime (if hot flushes are most bothersome at night) or during the day. Suggested dosage: Days 1-3: 300mg once daily, days 4-6: 300mg twice daily, days 7-28: 300mg three times daily. Gabapentin may cause dizziness and increased appetite. Gradually increasing the dose may help reduce these side effects. Gabapentin has similar effects to venlafaxine (Bordeleau et al., 2010; Loprinzi et al., 2010; Shan et al., 2020).

###### Oxybutynin

Oxybutynin is commonly used to treat overactive bladder and urinary incontinence. Placebo- controlled trials have shown that 73% of patients had fewer vasomotor symptoms after 12 weeks of treatment with oxybutynin 15 mg once daily (Simon et al., 2016). Dry mouth was reported by 52.1% of participants, and 6.8% discontinued treatment because of dry mouth. Other studies suggest that there is an effect at lower doses with fewer anticholinergic side effects (headache, diarrhoea, confusion): 2.5 mg twice daily or 5 mg twice daily reduced vasomotor symptoms by 60% and 77%, respectively (Leon-Ferre et al., 2020).

###### Neurokinin receptor antagonists

Elinzanetant is a non-hormonal dual neurokinin-1,3 receptor antagonist manufactured by Bayer. Placebo-controlled trials have already shown that this drug is safe and effective in reducing vasomotor symptoms in menopausal women without (a history of) breast cancer (Simon et al., 2023; Trower et al., 2020). Despite NK1,3 receptor antagonist studies show no major adverse events, caution is warranted concerning potential severe effects relating to liver toxicity. It is described in literature that patients experienced transient increases in liver transaminases, however no signs of liver toxicity were witnessed (Hassan et al., 2023). A randomised phase III study is currently underway to evaluate the effect in women with (a history of) breast cancer.

Fezolinetant is another neurokinin-3 receptor antagonist, recently approved by the United States Food and Drug Administration for moderate to severe hot-flushes in women with no history of breast cancer (Lederman et al., 2023). Fezolinetant 30 and 45mg were found to be efficacious and well tolerated for treating moderate to severe VMS associated with menopause (Johnson et al., 2023). Moreover, The North American Menopause Society (NAMS) has recently stated that the Fezolinetant can be reserved to use for alleviating the hot-flushes in the patients with oestrogen- dependent cancers or cardiovascular disease. The NAMS has also warned healthcare professionals to be well informed about non-hormonal treatment options for reducing VMS that are supported by the evidence (The Nonhormone Therapy Position Statement of The North American Menopause Society Advisory, 2023).

#### Systemic hormonal treatment

Breast cancer is a contraindication for the use of Systemic Replacement Therapy (HRT) due to the increased risk of recurrence in both the ipsi- and contralateral breast. This statement is based on the HABITS (Hormonal replacement therapy after breast cancer - is it safe?) trial (Holmberg et al., 2004). This randomised controlled trial enrolled women treated for stage II breast cancer and randomised them between HRT and non- hormonal treatment. The trial was stopped early due to significantly more recurrences in the HRT group (26 in the HRT group, 8 in the non-HRT group) after a median follow-up of 2.1 years (HR, 3.5; 95% CI, 1.5-8.1) and after 4 years the HR became 2.4 (95% CI, 1.3- 4.2) (Holmberg et al., 2008).

A second randomised controlled trial evaluating HRT after breast cancer is the Stockholm Randomised Trial. The trial included postmenopausal women who had undergone primary surgical treatment for histologically confirmed breast cancer. Patients were randomised to receive treatment with or without HRT for 5 years. A 10-year follow-up study concluded that there was no difference in the number of breast cancer-related events (local recurrence, distant metastasis, primary contralateral breast cancer or new primary malignancy) in the two groups (60 in the HRT group, 48 in the control group, HR = 1.3, 95% CI 0.9-1.9). However, there was an increased number of recurrences in the contralateral breast (14 in the HRT group and 4 in the control group, HR = 3.6, 95% CI 1.2-10.9; p = 0.013) (Fahlen et al., 2013).

The LIBERATE trial (Livial ® Intervention following Breast cancer: Efficacy, Recurrence, and Tolerability Endpoints) evaluated the effect of tibolone after breast cancer and concluded that tibolone was associated with an increased risk of recurrence (HR = 1.64, 95% CI 0.99-2.72) (Sismondi et al., 2011; Kenemans et al., 2009).

A meta-analysis by Mudhune et al. investigated whether the safety of HRT in women with (a history of) breast cancer differs according to their age at diagnosis (younger/older than 50 years). They concluded that there is sufficient evidence to suggest that HRT increases the risk of recurrence in women older than 50 years, but that this is unlikely to affect mortality (Mudhune et al., 2019).

A systematic review and meta-analysis on the safety of systemic HRT in breast cancer survivors showed a significant increase of recurrence for HRT (HR 1.46, 95% CI 1.12-1.91, p = 0.006). Subgroup analysis revealed that this increase is only significant for hormone receptor-positive cancers and not for hormone receptor-negative cancers (HR 1.8, 95% CI 1.15-2.82, p = 0.010; versus HR 1.19, 95% CI 0.80-1.77, p = 0.390) (Poggio et al., 2022).

Lupo et al. (2015) proposed a stepwise algorithm for the treatment of vasomotor symptoms, starting with non-hormonal therapy. If there is insufficient improvement, a comprehensive discussion should follow regarding the current evidence for and against HRT, and ultimately after shared decision-making, a decision can be made to use HRT.

BRCA ½ mutation carriers after risk-reducing salpingo-oophorectomy (RR-BSO) are a patient population exposed to early menopause. HRT in management of VMS in these women was not found to increase breast cancer risk in retrospective and longitudinal studies and meta-analysis. Several guidelines have recommended the use of HRT until the age of 45-50 years for healthy BRCA carriers following RR-BSO. Still, patients and healthcare providers appear to be reluctant to us HRT. A study by Armon et al. found that physician’s recommendation is a powerful determinant in favour for HRT use and failure to discuss treatment options may be perceived by patients as an indirect recommendation against treatment. The need for the physician to discuss HRT safety and its importance for women’s health and well-being is paramount (Armon et al., 2023).

### Aromatase inhibitor-induced arthralgia

Depending on the receptor status of the breast cancer and the age at diagnosis, endocrine therapy may consist of an aromatase inhibitor (AI). AI’s are used to block the activity of aromatase, an enzyme that the body uses to make oestrogen in the ovaries and other tissues. AI’s have been shown to improve survival and reduce the risk of recurrence (Early Breast Cancer Trialists’ Collaborative, 2015). However, this treatment is associated with significant side effects. Approximately 50% of breast cancer patients receiving adjuvant aromatase inhibitor therapy develop symptoms known as aromatase inhibitor- induced arthralgia (Hershman et al., 2018). Because of the significant impact on the quality of life of these patients, this also leads to poor adherence to these drugs.

#### Supplementation

Randomized trials on the effects of vitamin D and omega-3 fatty acids did not show a significant improvement in joint symptoms (Lustberg et al., 2018; Roberts et al., 2022). A randomised trial of turmeric extract and its effect on aromatase inhibitor-induced arthralgia is ongoing.

#### Body weight and physical exercise

A systematic review and meta-analysis suggest a protective effect of a BMI between 25-30 on the development of arthralgia (OR 0.33, 95% CI 0.14- 0.74) (Beckwee et al., 2017). Randomised trials show a significant improvement in aromatase inhibitor-induced arthralgia in patients who combine aerobic activity with resistance training (Irwin et al., 2015; Baglia et al., 2019).

#### Acupuncture

A meta-analysis suggests that acupuncture is a safe non-pharmacological option capable to significantly reduce arthralgia (Chen et al., 2017). However, one randomised trial suggests that there is a significant difference in favour of acupuncture compared with sham acupuncture or no acupuncture, but it is unclear what the clinical relevance of the improvement is (Hershman et al., 2018).

#### Duloxetine

A multicentre, randomised, placebo-controlled trial found significant improvement after 12 weeks of the SNRI duloxetine (Henry et al., 2018).

### Sleeping disorders

The incidence of sleep problems in women with breast cancer is 60-90% but varies between studies depending on study design and assessment methods (Weng et al., 2021). Most breast cancer patients experience insomnia or sleeplessness. The impact of insomnia on quality of life, pain symptoms, vasomotor symptoms and oncological recurrence is well documented (Fiorentino et al., 2011; Mansano-Schlosser and Ceolim, 2017; Marinac et al., 2017).

#### Sleep hygiene

The American Centre for Disease Control and Prevention lists some sleep habits on its website, including an active lifestyle, exposure to sunlight, consistent bedtime, and wake-up routines, relaxing with a book or bath before bed, keeping a quiet and dark bedroom at a comfortable temperature, and avoiding screens, large meals, caffeine and alcohol before going to bed.

#### Melatonin

A randomised, double-blind, placebo-controlled trial found a significant improvement in subjective sleep quality with no significant side effects. However, there was no difference observed in the incidence of depression or menopausal symptoms (Chen et al., 2014).

#### Cognitive-behavioural therapy, Tai Chi, and acupuncture

Arico et al. (2016) suggests that cognitive- behavioural therapy is an effective treatment for insomnia in breast cancer patients, with improvements in mood, overall and physical fatigue, and quality of life. A randomised trial by Irwin et al. compared cognitive behavioural therapy with Tai Chi and found that Tai Chi was statistically non-inferior to cognitive behavioural therapy, with robust improvements in sleep quality in the short term (3 and 6 months) and long term (15 months) (Irwin et al., 2017). Garland et al. (2017) concluded from their randomised controlled trial in 58 breast cancer survivors that electroacupuncture was as effective as gabapentin in improving sleep latency and efficiency.

### Vaginal dryness and dyspareunia

#### Sexual counseling, psychosexual education and couples therapy

Lack of lubrication is usually the cause of dyspareunia. Couples should be advised that lubrication can be improved through the use of lubricants, but also through adequate sexual arousal and stimulation. Although all postmenopausal women experience vaginal atrophy, only 20-30% experience dyspareunia, and vaginal dryness during intercourse. More recent studies suggest even higher percentages of bothersome dyspareunia as women don’t disclose this complaint. Lower oestrogen levels lead to a significant reduction in vaginal blood flow in postmenopausal women compared with premenopausal women. However, this significant difference in vaginal blood flow between postmenopausal and premenopausal women disappears after erotic stimulation (Alvisi et al., 2019). The increased vaginal blood flow after erotic stimulation results in increased lubrication. Dyspareunia and vaginal dryness during intercourse are largely due to inadequate sexual arousal and stimulation. Cognitive behavioural therapy, with or without exercise, has also shown significant improvement in urogenital symptoms (Duijts et al., 2012).

#### Topic vaginal non-hormonal treatment

Due to adjuvant endocrine treatment, chemotherapy and/or premature ovarian failure, women with (a history of) breast cancer often experience symptoms of the genitourinary syndrome of menopause. Symptoms of the genitourinary syndrome may include vaginal dryness, irritation, itching, recurrent infections, urinary urgency, and dyspareunia (NAMS, 2020; Perez-Lopez et al., 2021).

Iatrogenic ovarian suppression, tamoxifen and aromatase inhibitors can lead to vulvovaginal atrophy, which in turn can contribute to dyspareunia, reduced sexual function and libido. Studies have shown that these symptoms are often more severe and prolonged than those experienced during natural menopause. This can have a significant impact on adherence to adjuvant anti-hormonal therapy. It is therefore important to actively ask about these symptoms and, if they are present, to try to treat them as effectively as possible.

Treatment should be individualised and there is no one-size-fits-all approach. Non-hormonal options for menopausal genitourinary syndrome include daily use of vaginal moisturising creams, lubricants during sexual activity, topical application of xylocaine gel, pelvic floor therapy and dilators/vibrators (Perez-Lopez et al., 2021). The effect of laser therapy on vulvovaginal dryness is still under investigation. A systematic review that looked specifically at its effect in women with (a history of) breast cancer concluded that so far there are only small non-randomised trials suggesting an improvement (Knight et al., 2019). Mension et al. (2021) provided a systematic review of treatment options in breast cancer survivors and states that the CO2 laser appears to be effective in genitourinary syndrome of menopause, however data on safety is lacking. More specifically, the lack of objective measurements of vaginal mucosa changes before-after treatment, and no variables related to breast cancer recurrence or serum oestradiol measurements were studied.

Dynamic quadripolar radiofrequency (DQRF) is a novel technique that is also rapidly gaining attention. A systematic review showed improvements in vulvovaginal aesthetics, sexual satisfaction and relief of symptoms caused by vulvovaginal atrophy (Elbiss et al., 2022). All the studies to date have been conducted by people affiliated with the Novavision Group, the company behind DQRF. The technique has not been studied in women with (a history of) breast cancer.

#### Topic hormonal treatment

Topical oestrogens may be considered for women with (a history of) breast cancer who present with genitourinary symptoms when vaginal non- hormonal preparations do not provide adequate relief (Villa et al., 2020; Jain et al., 2020; Hirschberg et al., 2020; Mazzarello et al., 2015). There are no large randomised controlled trials demonstrating the safety of topical oestrogen therapy in breast cancer patients. However, several observational studies have shown no significant difference in recurrence. Some studies suggest that small amounts of oestrogen are absorbed systemically, and this could interfere with the working of AI’s, as they suppress plasma oestradiol levels. An observational study among anti-hormone users (tamoxifen or AI) showed no increase of recurrence after the use of vaginal oestrogens during follow-up period of 3.5 years (Le Ray et al., 2012). The use of ultralow-dose 0.005% estriol vaginal gel on the other hand do not significantly influence oestrogens, FSH, and LH levels in women with breast cancer receiving AI (Sanchez-Rovira et al., 2020). A randomised trial suggests a significant improvement in signs of vulvovaginal atrophy with the use of ultra- low dose 0.005% estriol vaginal gel compared with placebo (Hirschberg et al., 2020). There are no studies on long-term results or the risk of recurrence.

Vaginal dehydroepiandrosterone (DHEA), estriol and oral ospemifene are alternatives to local oestrogen therapy when non-hormonal measures do not provide adequate relief. Studies have shown that 6.5 mg of vaginal DHEA significantly improved genitourinary symptoms. DHEA is a precursor of androgens and did not cause a significant increase in blood levels of oestrogen and androgens in studies of postmenopausal women. However, there was a small increase in total blood testosterone, the significance and relevance of which is unknown. More studies are needed to establish safety, particularly in patients with breast cancer or a history of breast cancer.

Ospemifene is a SERM developed for the treatment of genitourinary symptoms and is not thought to have any estrogenic effects on the breast. However, it is not yet approved for use in breast cancer patients because there are no studies to prove its safety. Its use is not recommended by international guidelines (Barton et al., 2018; Simon et al., 2018; Cagnacci et al., 2020; Goldstein et al., 2014).

A randomised trial is currently underway to investigate the effect of four treatments (oestrogen, dehydroepiandrosterone, moisturisers, and a combination of oestrogen and probiotics) on vulvovaginal atrophy in breast cancer survivors (Vergauwen et al., 2023).

### Osteopenia and osteoporosis

#### Lifestyle interventions, calcium, and vitamin D intake

Adjuvant treatment (in the form of chemotherapy, GnRH agonists, or AIs) can lead to bone loss, potentially resulting in osteoporosis and fractures (Go et al., 2020). The prevention and treatment of osteoporosis in women with a history of breast carcinoma are similar to those in women without breast cancer (Ramin et al., 2018). The first step is to identify risk factors and reduce them where possible. This includes smoking cessation, limiting alcohol intake, adequate physical activity and fall training/prevention (Ramin et al., 2018). Secondly, an adequate daily intake of calcium and vitamin D is advised. The International Osteoporosis Foundation recommends a daily intake of 1200 mg of calcium and 800–1000 IU of vitamin D for postmenopausal women (Hadji et al., 2017). ESMO recommends a calcium-rich diet, moderate physical activity, and daily 1000- 2000 IU of vitamin D3 (Coleman et al., 2020). If dietary calcium intake is insufficient, a calcium supplement of 500-1000 mg is recommended.

#### Bisphosphonates

Bisphosphonates, including zoledronic acid, are potent inhibitors of bone resorption. They also have anti-tumour activity, such as inhibiting osteoclastic bone resorption and osteoblastic proliferation, slowing the progression of bone abnormalities, and reducing the risk of fractures and bone pain. ESMO recommends bisphosphonates for women with early breast cancer who have low oestrogen levels and for women with treatment-related bone loss. Bisphosphonate use can cause osteonecrosis of the jaw (ONJ) in 1 to 10% of patients. Important preventive measures include prophylactic dental examinations, good oral hygiene, and regular dental visits. Bisphosphonates are safe for long- term use and are effective in reducing the risk of bone-related events, such as pathological fractures and spinal cord compression. This is particularly important because the incidence of bone-related events is highest in breast cancer patients compared with all other cancer patients (Coleman et al., 2020).

## Further research

It is clear from the literature that the effects of cognitive behavioural therapy, Tai Chi and education are significant in the reduction of menopausal symptoms. However, existing studies have mainly been conducted in smaller groups and larger multicentre trials are needed. Ongoing trials are also investigating systemic non-hormonal drugs to relieve menopausal symptoms in women with (a history of) breast cancer, such as elinzanetant.
